# Importance of sample selection and generalisability in scientific methodology

**DOI:** 10.55730/1300-0144.6089

**Published:** 2025-08-06

**Authors:** Egemen ÜNAL, Şefik YURDAKUL, Okan MADEN

**Affiliations:** Department of Public Health, Faculty of Medicine, Ankara Yıldırım Beyazıt University, Ankara, Turkiye

Dear Editor,

A well-planned methodology in scientific research is essential both for obtaining accurate results and for adequately representing the target population. In studies aiming for generalisability, a representative and sufficiently large sample size is a fundamental prerequisite [1].

In cross-sectional studies, selecting a sample that accurately represents the population allows the findings to be generalised. If a nonprobabilistic sampling method is used, the sample may not accurately represent the population, making generalisation inappropriate; therefore, such studies should refrain from claiming that their findings are generalisable [2]. Studies assessing health conditions must use proper sampling to reflect the community’s health status accurately. Inadequate samples may distort the true prevalence and lead to incorrect decisions and interventions.

When calculating the sample size of population-based prevalence studies, referencing results from previous studies with similar characteristics or using the expected prevalence from a pilot study can guide appropriate sample size determination [3].

A common method involves using 384 as a minimum sample size, based on an assumed 50% frequency, regardless of population size. This approach results in identical sample sizes for vastly different populations-such as 10,000 and 10 million-which can lead to inaccurate selection, especially in large populations. To improve reliability in such studies, multi-stage and probability sampling should be employed, along with accurate estimation of intraclass correlation and design effect [4–6]. A well-known national example is the Turkey Household Health Survey 2023, which employed a stratified, multi-stage, and probability-based sampling method to generate representative data on the population’s health status and risk factors [7].

The method of participant selection is as important as the sample size in research. Inclusion criteria must be defined precisely and described clearly and understandably [8]. For example, in a study aiming to represent children under 5 years old living in Ankara, selecting participants from only one district or failing to detail the inclusion criteria in the methodology section may indicate selection bias [9]. Studies focusing on relatively small and hard-to-reach populations are often unreliable due to increased sampling error [4].

The primary concern in sampling for scientific research is the risk of bias resulting from inappropriate sample selection. For instance, in a prevalence study conducted in a village, if the minimum sample size is set at five hundred but only individuals who are encountered and agree to participate are included—without ensuring representativeness—others are denied an equal chance of participation. Consequently, the findings cannot be generalised to the entire village, and the true prevalence may be misrepresented. Accordingly, increasing attention is being paid to study design and potential sources of bias when evaluating published research [10].

In conclusion, methodology in scientific research should be carefully structured. In particular, sample selection is critical for the generalisability of findings; inadequate or flawed sampling may undermine the study’s internal and external validity. Therefore, researchers must follow a rigorous methodological framework throughout every stage of the study and report it clearly.
